# Development of an Analytical Protocol for Determination of Cyanide in Human Biological Samples Based on Application of Ion Chromatography with Pulsed Amperometric Detection

**DOI:** 10.1155/2017/7157953

**Published:** 2017-11-21

**Authors:** Ewa Jaszczak, Marek Ruman, Sylwia Narkowicz, Jacek Namieśnik, Żaneta Polkowska

**Affiliations:** ^1^Department of Analytical Chemistry, Faculty of Chemistry, Gdańsk University of Technology, Narutowicza Str. 11/12, 80-233 Gdańsk, Poland; ^2^Faculty of Earth Sciences, University of Silesia, Będzińska 60, 41-200 Sosnowiec, Poland

## Abstract

A simple and accurate ion chromatography (IC) method with pulsed amperometric detection (PAD) was proposed for the determination of cyanide ion in urine, sweat, and saliva samples. The sample pretreatment relies on alkaline digestion and application of Dionex OnGuard II H cartridge. Under the optimized conditions, the method showed good linearity in the range of 1–100 *μ*g/L for urine, 5–100 *μ*g/L for saliva, and 3–100 *μ*g/L for sweat samples with determination coefficients (*R*) > 0.992. Low detection limits (LODs) in the range of 1.8 *μ*g/L, 5.1 *μ*g/L, and 5.8 *μ*g/L for urine, saliva, and sweat samples, respectively, and good repeatability (CV < 3%, *n* = 3) were obtained. The proposed method has been successfully applied to the analysis of human biological samples.

## 1. Introduction

Toxic substances, such as cyanide ions, are present in the environment as soluble salts (e.g., sodium salt) and insoluble compounds (e.g., mercury) [1]. They can be metabolized and excreted from the body with biological fluids in their unchanged form or as metabolites. The process of cyanide's metabolism in the living organism can take place in various ways and its products are extracted through the lungs and with body fluids. Much of the cyanide, which is absorbed into the body through the respiratory tract, gastrointestinal tract, and skin, is detoxified in the liver due to the thiosulfate sulfotransferase enzyme present in the mitochondria of the liver. This reaction transforms cyanide into the 200-fold less toxic thiocyanate, which is easily soluble in water and excreted from the organism together with body fluids. The remaining cyanide ions are oxidized into methanoate and carbon dioxide [2]. A small amount of cyanide ions links with cysteine and creates 2-iminotiazolidyno-4-carboxylic acid [3]. These processes depend on the health of the organism and the physicochemical properties of the absorbed substance [4].

The presence of cyanide in the body leads to inhibition of cell respiration, as a result of the merger of cyanide ion with trivalent iron cation of cytochrome a3, an integral component of cytochrome oxidase, located in the mitochondria of the liver. Hypoxia results in disorder in functioning of all cells; however, the most sensitive to the toxic effects of cyanide are those tissues with the fastest oxygen metabolism like cardiac muscle and the brain [5]. Therefore, the analysis of cyanide in samples with different matrix compositions becomes a very important issue. However, biological samples have challenging sample matrices with varying sample preparation techniques [6–8]. The sample preparation method depends on the analytical technique. Most methodologies of preparing samples for the determination of total cyanide content consist of adding sodium hydroxide to pH greater than 12, which results in trapping the volatile forms of cyanides and then in carrying out the distillation operation. Spectrophotometric techniques [9–11], which have been used for many years, are now mostly replaced by chromatographic ones (both GC [12, 13] and LC [14]), which are characterized by higher selectivity and sensitivity, while capillary electrophoresis [15] is rarely used.

The aim of this work is to carry out fast and accurate determination of cyanide ion in biological materials using IC-PAD. To the best of our knowledge, no work has been performed so far to evaluate the status of cyanide ion in biological materials using IC-PAD detection. The proposed method was applied to the determination of cyanide ion in biological materials of a group of smokers and passive smokers. Moreover, cyanide ions may be biomarkers of exposure to tobacco smoke components, so the proposed method can also be applied for medical use.

## 2. Materials and Methods

### 2.1. Chemicals and Reagents

It is essential to use high-quality degassed water and sodium hydroxide (50% solution) for the eluent. All reagents and chemicals were purchased from Sigma-Aldrich (Missouri, USA). Deionized water was obtained from Millipore Gradient A10 (resistivity: 18.2 Ωcm at 25°C) water purification system (Millipore, Bedford, USA).

### 2.2. Instrumentation

**Figure 1:**
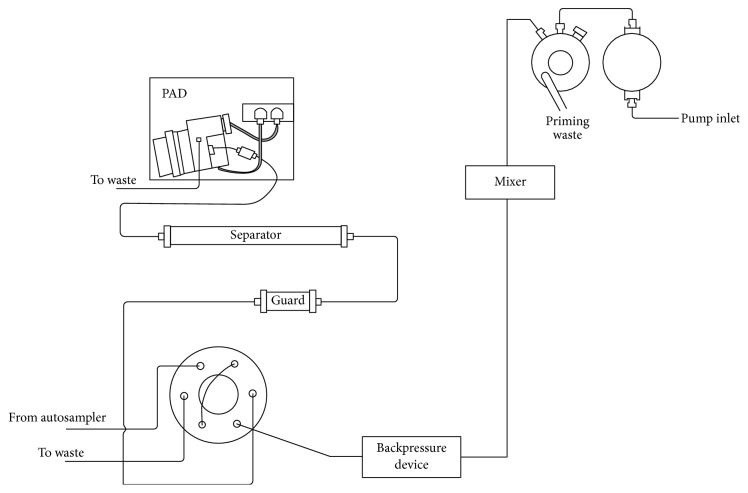
Schematic diagram of the instrumental setup.

Analyses were performed on an IC-PAD system consisting of a Dionex ICS 3000 system (Dionex, Sunnyvale, CA, USA) equipped with a gradient pump, autosampler, column oven, degasser, and a pulsed amperometric detector (Ag working electrode, Ag/AgCl reference electrode) (Figure 1). The PAD waveform was as follows: (0 s, −0.1 V), (0.2 s, −1 V), (0.9 s, −0.1 V) (0.91 s, −1 V), (0.93 s, −0.3 V), (1 s, −0.3 V). Separation was performed by a Dionex IonPac AS15 anion-exchange column (Thermo Scientific, 4 × 250 mm). The analytical column was protected by a Dionex IonPac AG15 anion-exchange guard column (Thermo Scientific, 4 × 50 mm). The column was equilibrated with the use of the 63 mM sodium hydroxide eluent. The eluent flow rate was set at 1 ml/min. Injection volume was 40 *μ*L. All measurements were made at 30°C. The column backpressure was approximately 1100 psi (7.58 MPa). All samples were analyzed at a minimum in triplicate. The analytical data were acquired and analyzed using the Chromeleon software (version 6.8).

### 2.3. Collection and Preparation of Biological Samples

The research was based on biological material of human origin; it required the consent of the Bioethics Committee. This was granted on 8 January 2015 (number NKEBN/571/2014-2015). Biological samples of urine, saliva, and sweat were collected from three groups of donors:

- Control group: people who do not actively or passively smoke tobacco
- Active smokers' group: people who actively smoke tobacco
- Passive smokers' group: people exposed to components of environmental tobacco smoke

**Figure 2:**
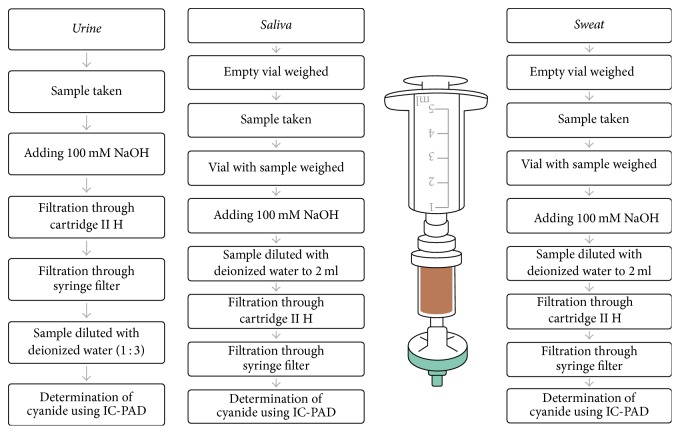
The process of sample preparation for analysis.

Biological samples should be prepared to prevent cyanide degradation and cyanide should be determined as soon as possible. Free cyanide present at neutral pH will volatilize to hydrogen cyanide. Also, oxidizing agents decompose cyanide. Moreover, because of these issues, 0.5 mL of 100 mM NaOH solution was added to pH > 12 (Figure 2). Sample solutions were transferred through Dionex II H cartridge (1 cc, Thermo Scientific) and filtered through a syringe filter before sample injection. These samples were tested immediately. The cartridge removes high levels of alkali and alkaline earth metals and cationic transition metals. Until the analysis, samples of urine, saliva, and sweat were stored in polypropylene tubes, protected from light, at a temperature of approx. −15°C.

### 2.4. Method Validation

Blank samples (biological samples from the control group) were used in order to validate the method. 1 mg/L cyanide was prepared with 0.377 mg of sodium cyanide in 100 mM sodium hydroxide. Calibration curves were prepared by adding an appropriate amount of stock solution to the blank urine, resulting in final concentrations of 1, 2, 3, 5, 7, 10, 30, 50, 70, and 100 *μ*g/L cyanide. Saliva and sweat samples were prepared similarly to urine. Working solutions were prepared in a concentration range of 5–100 *μ*g/L. Measurements were performed in triplicate for each spiked sample.

**Figure 3:**
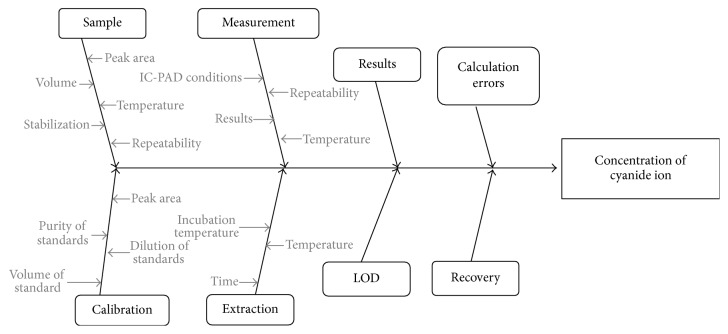
The Ishikawa diagram presenting the influence of parameters on the analytical process for the determination of cyanide ion in a biological sample.

The procedure for determining cyanide ions in biological samples was validated to ensure the appropriate level of quality control and quality assurance of measurements. The validation process of the analytical method requires determination of selectivity, repeatability, and linearity as well as limits of detection and quantification. Most items are well known and do not need explanation. The linearity of calibration curves was assessed by plotting the peak area against the concentration of the standard and expressed by the correlation coefficient (*R*^2^). The limit of detection (LOD) was determined on the basis of the numerical value of the signal-to-noise ratio in samples with very low analyte concentrations. For that, the level of noise was assessed by measuring the peak-to-peak noise per min for 5 min. The limit of detection value was the concentration producing a signal-to-noise ratio based on the chromatogram obtained for blank sample, whereas the limit of quantification (LOQ) is 3x the LOD. The most common measure of precision is standard deviation, relative standard deviation, or coefficient of variation (CV). The CV content of the analyte should not be greater than 3%. The precision of this procedure is expressed as a numerical value of the coefficient of variation calculated for results of three simultaneous analyses. For that, the standard deviation (SD) value was divided by the average area of the peak. A known amount of analytes (*s*) and standards was added to one sample while another sample was only spiked with standards at the same concentration level. After completing the analysis with the sample without the added analytes (*x*) and the sample containing added analytes (*x* + *s*_*i*_), the recovery was calculated using the following equation: recovery = (*x* + *s*_*i*_) − *x*/*s* [18]. Sources of uncertainty related to the determination of cyanide ions in biological samples were presented in the form of a fishbone diagram (Ishikawa diagram) (Figure 3). 

## 3. Results and Discussion

### 3.1. Validation Parameters

**Figure 4:**
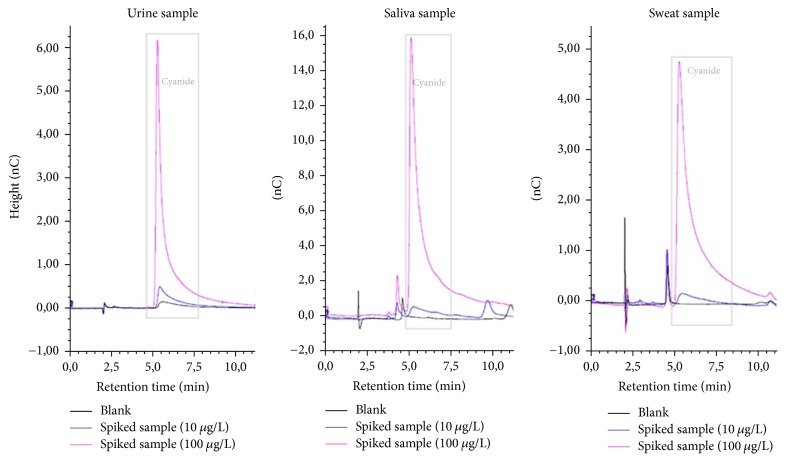
Chromatograms obtained from blank and spiked biological samples.

Sulfides, thiocyanates, and nitrates were previously reported as interference to the quantitative determination of cyanide [19, 20]. The sample preparation process was used to determine total cyanide by decomposing the stable cyanide-metal complexes. The developed analytical procedure ensures the possibility of quantitative determination of cyanide. No changes were noted during the retention time of the cyanide ion in the analysis of the properly prepared samples, which is an indicator of generally good laboratory practice and reliable instrumentation (Figure 4).

**Figure 5:**
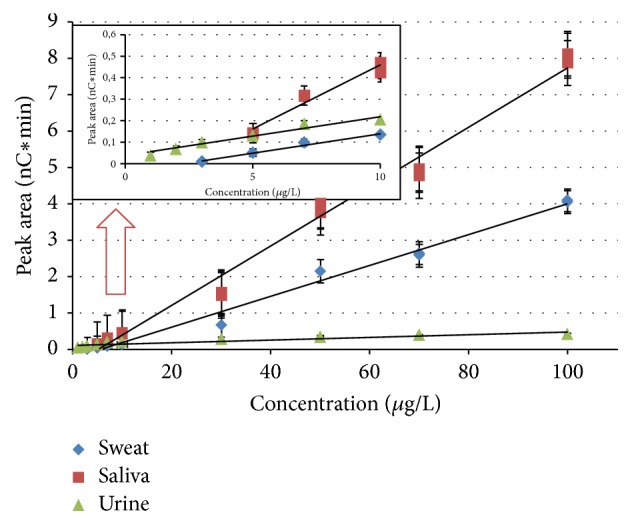
Ion chromatography calibration curves for cyanide ion analyzed in biological samples.

Calibration curves were analyzed and demonstrated to be linear over a concentration range of 3 to 100 *μ*g/L for sweat, 5 to 100 *μ*g/L for saliva, and 1 to 100 *μ*g/L for urine with correlation coefficients of, respectively, 0.993, 0.994, and 0.992 (Figure 5 and Table 1). Average recovery results were higher in saliva (113%) than in sweat (88%) or urine (80%) samples, but recoveries between 80 and 120% are usually acceptable. The results of the reproducibility tests by triplicate injection are acceptable, below 3% RSD value. The known addition of cyanide standard did not result in any duplex peaks, supporting the cyanide identification.

### 3.2. Application

**Figure 6:**
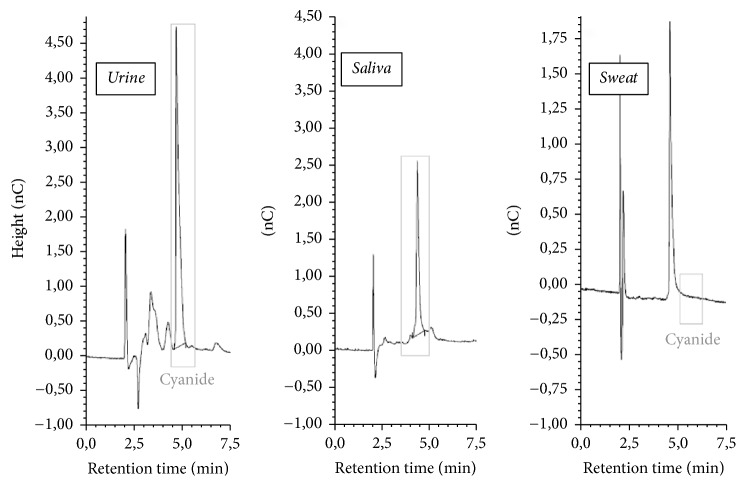
Examples of chromatograms of tested smoker's urine (12.45 *μ*g CN^−^/L) and saliva (7.37 *μ*g CN^−^/L) and nonsmoker's sweat (<LOD) samples.

The method was successfully applied to the determination of cyanide ion in human biological samples (Figure 6). Furthermore, the contents of cyanide ion in diluted samples were calculated via the standard equation in Section 3.1, with the concentrations in Table 2. Considering the achieved results, no cyanide ions were detected in sweat samples, but it was hypothesized that this was due to a very sporty lifestyle and the specially tailored donor diet. The donors who gave sweat samples were Thai boxing players. The current research allows estimating the level of a biomarker of exposure to environmental tobacco smoke constituents in samples of biological materials derived from selected donors.

Compared to other procedures used for the determination of cyanide ion (Table 3), the developed sample preparation procedures for preparation of biological (urine, saliva, and sweat) sample materials are simple and are not time-consuming [2]. Moreover, it is possible to use a small amount of sample (approx. 1 mL), which is a particular advantage in relation to biological materials samples, as it is typically difficult to obtain large quantities. The achieved results show that the proposed technique can be used to monitor cyanide concentrations in biological samples.

## 4. Conclusions

In the present work, IC-PAD was developed and fully validated to quantify cyanide in human biological samples. The method used at the stage of preparing biological samples has many advantages, for example, the placement in extraction cartridges of a very small amount of sample and the use of water solutions as a solvent. Furthermore, the validated method was successfully applied to analyze urine, sweat, and saliva samples from volunteers. The conducted studies can confirm the influence of tobacco smoking on the level of concentration of cyanide ion. Urine, saliva, and sweat samples are good biological materials for the evaluation of the exposure to cyanide. Cyanide ion is a very good biomarker of the exposure to the ingredients of tobacco smoke because it has a relatively long half-life (2 h) and differences between the concentrations of those ions among the various donor groups can be observed [2, 19]. The highest concentrations of cyanide ion were detected in the group of actively smoking donors.

## Figures and Tables

**Table 1:** Parameters of the IC-PAD method for the determination of cyanide ion.

Sample	Linear range [*µ*g/L]	Curve pattern	*R*	SD	CV (%)	Recovery [%]	LOD [*µ*g/L]
Urine	1–10	*y* = 0.019*x* − 0.034	0.983	0.001	0.42	62	1.8
	1–100	*y* = 0.169*x* − 0.304	0.992	0.003	1.63	80	
Saliva	5–10	*y* = 0.059*x* − 0.133	0.988	0.007	2.48	104	5.1
	5–100	*y* = 0.082*x* − 0.423	0.994	0.05	1.84	113	
Sweat	3–10	*y* = 0.018*x* − 0.041	0.987	0.002	2.57	108	5.8
	3–100	*y* = 0.042*x* − 0.243	0.993	0.01	0.88	88	

**Table 2:** Comparison of the results of cyanide in biological samples.

Studied subjects	Sample	Mean concentration of cyanide [*µ*g/L]
Active smokers	Urine (*n* = 25)	23.76 ± 4.73
	Saliva (*n* = 11)	16.62 ± 5.12
	Sweat (*n* = 0)	—^a^

Passive smokers	Urine (*n* = 24)	11.52 ± 4.14
	Saliva (*n* = 14)	20.7 ± 2.39
	Sweat (*n* = 3)	<LOD

Nonsmokers	Urine (*n* = 15)	6.90 ± 2.85
	Saliva (*n* = 10)	16.65 ± 0.71
	Sweat (*n* = 6)	<LOD

^a^No data.

**Table 3:** Comparison of validation parameters for different methods of marking cyanides in biological materials samples.

	Sample	Linear range	*R*	LOD	Recovery [%]	Run time [min]	References
IC-PAD	Urine	1–100 *μ*g/L	0.992	1.8 *μ*g/L	80	25	Present work
	Saliva	5–100 *μ*g/L	0.994	5.2 *μ*g/L	113		
	Sweat	3–100 *μ*g/L	0.993	5.8 *μ*g/L	88		
GC-MS	Plasma urine	20–100000 *μ*g/L	0.998	40 *μ*g/L	90.58–115.56	25	[16]
Spectrophotometric method	Blood	—^a^	—^a^	60 *μ*g/L	—^a^	—^a^	[7]
GC-MS	Blood	0.02 *µ*M/L	—^a^	0.1 *µ*M/mL	80	—^a^	[12]
IC-UV/Vis	Blood	3.8–7690 pM/mL	—^a^	3.8 pM/mL	83	—^a^	[17]
Spectrophotometric method	Nasal discharge	—^a^	0.995	2 *μ*g/L	—^a^	—^a^	[11]

^a^No data.
